# Androgen deprivation modulates the inflammatory response induced by irradiation

**DOI:** 10.1186/1471-2407-9-92

**Published:** 2009-03-25

**Authors:** Chun-Te Wu, Wen-Cheng Chen, Paul-Yang Lin, Shuen-Kuei Liao, Miao-Fen Chen

**Affiliations:** 1 Department of Urology, Chang Gung Memorial Hospital, Linko, Taiwan; 2 Chang Gung University College of Medicine and Chang Gung Institute of Technology, Taiwan; 3 Department of Radiation Oncology, Chang Gung Memorial Hospital, Chiayi, Taiwan; 4 Department of Pathology, Chang Gung Memorial Hospital, Chiayi, Taiwan

## Abstract

**Background:**

The aim of this study was to determine whether radiation (RT)-induced inflammatory responses and organ damage might be modulated by androgen deprivation therapies.

**Methods:**

The mRNA and tissue sections obtained from the lungs, intestines and livers of irradiated mice with or without androgen deprivation were analyzed by real-time PCR and histological analysis. Activation of NF-kappa B was examined by measuring nuclear protein levels in the intestine and lung 24 h after irradiation. We also examined the levels of cyclooxygenase-2 (COX-2), TGF-β1 and p-AKT to elucidate the related pathway responsible to irradiation (RT) -induced fibrosis.

**Results:**

We found androgen deprivation by castration significantly augmented RT-induced inflammation, associated with the increase NF-κB activation and COX-2 expression. However, administration of flutamide had no obvious effect on the radiation-induced inflammation response in the lung and intestine. These different responses were probably due to the increase of RT-induced NF-κB activation and COX-2 expression by castration or lupron treatment. In addition, our data suggest that TGF-β1 and the induced epithelial-mesenchymal transition (EMT) via the PI3K/Akt signaling pathway may contribute to RT-induced fibrosis.

**Conclusion:**

When irradiation was given to patients with total androgen deprivation, the augmenting effects on the RT-induced inflammation and fibrosis should take into consideration for complications associated with radiotherapy.

## Background

Although radiotherapy is an important cancer treatment modality, the cell killing induced by radiation is not tumor- or cell-type specific. Treatment of cancer patients with radiation can be significantly compromised by the development of severe acute and late damage to normal tissues. Normal tissue complications induced by irradiation differ depending on the target organ and cell types. Sequelae of pelvic RT include small bowel obstruction, enteritis, proctitis and radiation cystitis [1,2], and the lung is one of the most critical dose- limiting organs after thoracic RT. Recent studies have led towards a better understanding of the molecular mechanisms underlying radiation injury [3-5]. The response to radiation is dynamic and involves several mediators of inflammation and fibrosis that are produced by macrophages, epithelial cells and fibroblasts. Not surprisingly, pro-inflammatory cytokines are highly prominent among the panoply of molecules expressed in tissue after irradiation, and has been demonstrated to contribute to the significant complications associated with radiotherapy [5-7]. Since persistent accumulation and activation of immune cells is a hallmark of chronic inflammation, early manipulation of inflammatory responses could be useful for modification of the subsequent late effects [8,9].

In many respects, the tissue responses to irradiation mimic the cytokine storms generated by many other tissue damaging insults, such as hemorrhagic shock and sepsis. Hemorrhagic shock results in excessive production of pro-inflammatory mediators, which play a critical role in the development of multiple organ dysfunctions under such conditions. After hemorrhagic shock and sepsis, gonadal steroids have a significant effect on the maladaptive changes in immune cell function [10-12]. Gender-dimorphic immune and organ responsiveness have been reported [13,14]. Androgens might mediate the immunosuppression following trauma-hemorrhagic shock in males, whereas female sex steroids have immunoprotective properties after hemorrhagic shock and sepsis. Testosterone reportedly has key roles in fibrosis and wound healing via cell-specific and differential regulation [15,16]. Furthermore, a combination of hormone treatment and curative radiation treatment was often given to patients with prostate cancer and breast cancer [17,18]. Based on these reports, it triggers an unsolved issue; if the inflammation response and fibrosis induced by irradiation (RT) might be regulated by hormone manipulation through surgical or medical method. Therefore, the aim of the present study was to test the hypothesis that the inflammatory response and organ damage induced by irradiation can be modulated by androgen deprivation therapies including administration with flutamide (anti- androgen) and castration.

## Methods

### Mice, radiation and androgen deprivation (flutamide administration or castration)

**Figure 1:**
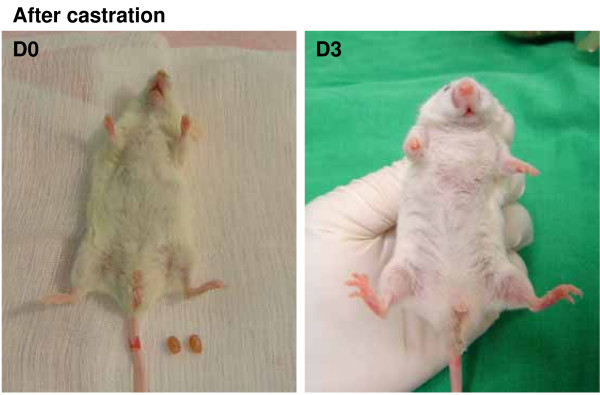
**Representative picture for mice after castration**.

BALB/c mice were purchased from the National Science Council, Taiwan. A total of 60 male mice aged between 6 and 8 weeks old were used in this study. The protocol of animal experimentation was approved by the Chang Gung Memorial Hospital Experimental Animal Committee. For irradiation, anesthetized mice were restrained in modified Perspex tubes and received 20 Gy total body irradiation by 6 MV X-rays from a linear accelerator. Unirradiated mice were subjected to the same conditions but were not exposed to the radiation source (sham-irradiation). For androgen deprivation treatment, the mice were divided into three groups: 1. C-mice, the mice without any androgen deprivation treatment; 2. F-mice, the mice with flutamide administration (the mice were injected twice intraperitoneally with flutamide (1 mg/g dissolved in DMSO), 12 h before irradiation and 2 h after irradiation); 3. S-mice, the mice receive androgen deprivation treatment by surgical castration (Figure 1). In addition, TGF-β1 has a central role in modulation of immune reaction. We proposed TGF-β1 contributed to the RT-induced fibrosis via the PI3K/Akt signaling pathways. To test the hypothesis, the mice were injected intraperitoneally with 200 ng TGF-β1 for 3 days before following experiments or treated with Wortmannin, a PI3K/Akt inhibitor (0.1 mM in DMSO), 12 h before irradiation.

### RNA isolation and real-time RT-PCR

At the indicated times after irradiation, Four mice from each group were sacrificed by cervical dislocation and the lungs, intestine and liver were dissected and stored at -80°C pending analysis. Specific Assay-on-Demand Gene Expression Assay mixes (including primer and Taqman MGB probes) for IL-1α/β, IL-6, TNF-α and TGF-β were used for real-time PCR analysis (Applied Biosystems, Foster, CA, USA). The first strand cDNA was amplified through 40 cycles (95°C for 15 s and 60°C for 1 min) with the TaqMan Universal PCR Master mix and the specific Assay-on-Demand Gene Expression Assay mix for each gene according to the manufacturer's instructions. To compare loading differences, a GAPDH primer was used as the internal control. Optimized PCR was performed on an iCycler iQ multicolor real-time PCR detection system. Significant PCR fluorescent signals were normalized to a PCR fluorescent signal obtained from the mean value of the sham-irradiated control mice. Two- sided log- rank analysis was used to assess statistical significance, and the association between discrete variables was tested using t-test.

### Assessment of myeloperoxidase (MPO) activity

MPO activities were determined in whole lung, liver and intestinal homogenates as described by Toth et al. [19]. Tissue samples were collected, frozen in liquid nitrogen and stored at -80°C pending analysis. The samples were sonicated on ice, then centrifuged at 12,000 g for 15 min at 4°C. Aliquots were divided into 180 μl phosphate buffer (pH 6.0) containing 0.167 mg/ml o-dianisidine dihydrochloride and 0.0005% hydrogen peroxide. The reactions were monitored by spectrophotometry at 460 nm (model 550, Bio-RAD, Hercules, CA) for 10 min.

### Histological analysis and immunochemical staining

Cellular aspects of inflammation were measured in lung and intestinal tissue samples using hematoxylin and eosin (H&E) and immunochemical staining. Treated and control mice were sacrificed by cervical dislocation 24 h after exposure to 20 Gy irradiation. Three mice for each group were used, and the lungs and intestines were fixed pre-autopsy with 10% formalin. Moreover, the lung and intestinal tissue samples from mice with Lupron treatment (with Lupron Depot (0.4 mg/Kg) at two weeks before irradiation) were also analyzed. For histological analysis, the tissues were fixed in 10% buffered formalin, paraffin-embedded and sectioned at an average thickness of 5 μm. The mounted sections were subjected to H&E and immunochemical staining. Briefly, samples were incubated overnight with goat anti-mouse TGF-β1 antibody (Santa Cruz Biotechnology, Inc., Santa Cruz, CA, USA) diluted 1:100 in 0.01 M RPMI at room temperature. After washing three times with PBS, the sections were incubated with biotinylated anti-goat IgG (1:100) for 10 min followed by peroxidase-avidin staining. Samples were washed with PBS, followed by addition of 3-amino-9 ethylcarbazole.

### Immunoblotting

For western blot analysis of whole cell extracts, cells were lysed in a lysis buffer and the nuclear and cytoplasmic proteins were separated using an NE-PER kit (Pierce, Rockford, IL, USA). Equal amounts of protein were loaded onto SDS-PAGE gels. After electrophoresis, the proteins were transferred to nitrocellulose membranes. The membranes were incubated with antibodies specific for TGF-β1 and COX-2 (Santa Cruz Biotechnology, Inc), followed by incubation with horseradish peroxidase-conjugated secondary antibodies. Signals were detected using enhanced chemiluminescence. To normalize the protein loading, the membrane was re-probed with mouse anti-r-tubulin antibody (1:1000).

### Electrophoretic mobility gel shift assays (EMSA)

Nuclear proteins were collected from BALB/c mice lung, intestinal and liver tissues 24 h after exposure to 20 Gy. Four murine lung tissues from each group were checked. The protein content was measured using the Bradford method. The DNA oligonucleotide used for NF-κB binding was 5'-AGTTGAGGGACTTTCCCAGGC-3', a sequence specific to NF-κB binding for mouse. The NF-κB binding activity was evaluated using a LightShift Chemiluminescent EMSA kit (Pierce, Rockford, IL, USA). Anneal oligo by mixing together equal amounts of the labeled complementary oligos and incubating the mixture for 1 h at room temperature. Nuclear extracts were incubated with the biotin-labeled DNA probe for 20 min at room temperature. The DNA-protein complex was separated from free oligonucleotides on a 5% polyacrylamide gel, transferred to a nylon membrane and cross-linked by UV. The membrane was incubated with streptavidin-horseradish peroxidase conjugate and detected by Enhanced chemiluminescence (Pierce, Rockford, IL, USA).

## Results

### Effects of androgen deprivation including castration and flutamide administration on the radiation-induced expression of pro-inflammatory cytokines

**Figure 2:**
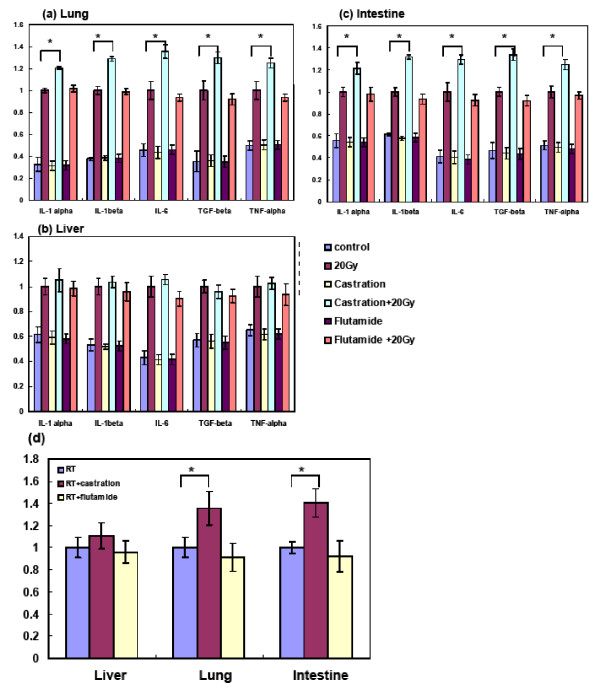
**Effect of androgen deprivation including flutamide and castration on pro-inflammatory cytokines in lung, liver and intestinal tissues**. (A-C) The mRNA levels of the cytokines TNF-α, IL-6, IL-1α/β and TGF-β were quantified by real-time RT-PCR. The results were normalized to the value of irradiated mice. The y-axis shows the RNA ratio of each target gene in lung (A), liver (B) and intestinal (C) tissue (control and irradiated mice with flutamide administration) divided by that in the irradiated mice (without flutamide administration). (D) Effect of flutamide on the MPO activities in lung, liver and intestinal tissues. The y-axis shows the ratio of MPO activity in irradiated tissues with flutamide administration or castration divided by irradiated tissues without androgen deprivation. Data are the mean ± SE. Columns, means of 3 separate experiments; bars, SD. * *P *< 0.05

Real-time RT-PCR was used to quantify the expression of cytokines induced by radiation and their changes after androgen deprivation. First, low expression levels were noted in unirradiated control mice and there were no obvious changes after androgen deprivation (unirradiated F-mice and S-mice). Irradiation (20 Gy) induced a significant increase in the mRNA levels of detected cytokines of the experimental groups compared with the unirradiated group at 24 h. Castration augmented the increase in pro-inflammation cytokines in the irradiated lung, intestine, but not in liver (Figure 2A–C). However, our data demonstrated that androgen deprivation by flutamide did not induce obvious changes on RT-induced inflammation. In addition, a significant increase in MPO activity was observed in the irradiated lung and intestine after castration (Figure 2D). Administration of flutamide did not significantly affect the MPO activity in irradiated mice.

### Effect of androgen deprivation on the radiation-induced inflammatory response by Histologic examination

**Figure 3:**
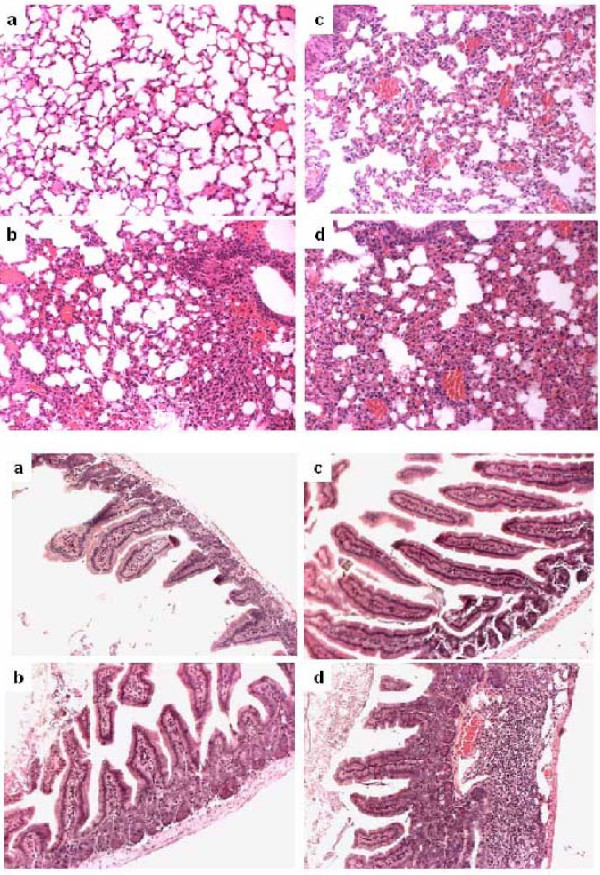
**Effects of flutamide administration and castration on the radiation- induced inflammation**. Lung and intestinal tissue sections were subjected to histologic analyses using H&E staining (Magnification ×200) were performed on lung (A) and intestinal (B) sections. Three mice from each group were examined. Representative slides are shown for (a) unirradiated control mice, (b) irradiated mice at 24 h after 20 Gy irradiation, (c) flutamide-treated mice 24 h after 20 Gy irradiation and (d) castrated mice 24 h after 20 Gy irradiation. Increased acute inflammatory infiltrate was observed in the interstitium was detected 24 h after irradiation. Duplicate experiments were performed for the analysis.

**Figure 4:**
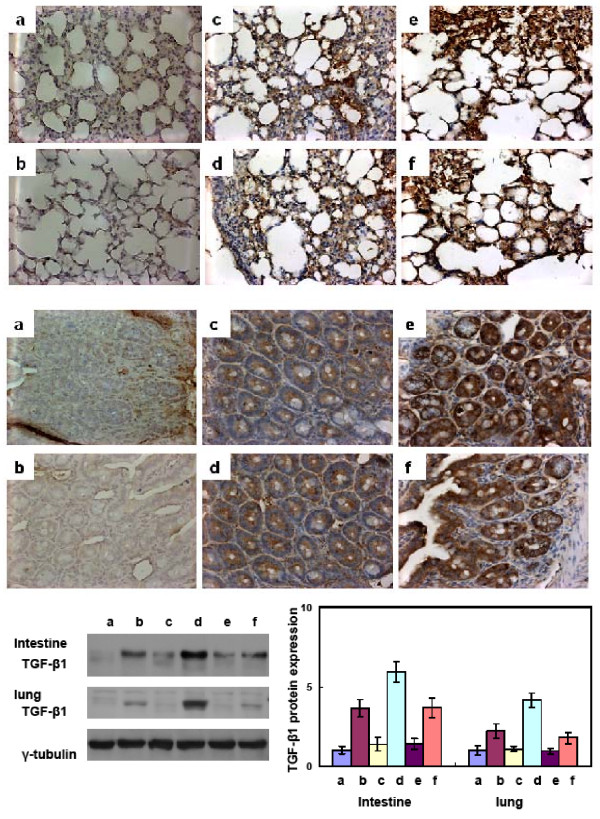
**Effects of flutamide administration and castration on the radiation- induced injury by immunochemical analysis**. Immunohistochemical analyses using an antibody against TGF-β (Magnification ×400) were performed on lung (A) and intestinal (B) tissues. Three mice from each group were examined. Representative slides are shown for (a) unirradiated control mice, (b) castrated mice without irradiation, (c) irradiated mice at 24 h after 20 Gy irradiation, (d) flutamide-treated mice 24 h after 20 Gy irradiation, (e) castrated mice 24 h after 20 Gy irradiation and (f) Lupron-treated mice 24 h after 20 Gy irradiation. Increased TGF-β immunoreactivity was detected 24 h after irradiation. Castration augmented the inflammatory response, whereas androgen deprivation by Flutamide administration had no significant effect. Duplicate experiments were performed for the analysis. (C) Expression of TGF-β1 protein in irradiated murine intestinal and lung tissue with or without androgen deprivation including castration and flutamide administration. (a, control; b, irradiation; c, castration alone; d, castration plus irradiation; e, flutamide administration; f, irradiation plus flutamide administration). (Y axis represents the relative protein level which is normalized by the protein level of TGF-β1 in control condition).

No significant lesions were observed in non-irradiated tissues from mice of each group. Increased levels of acute inflammatory infiltrate were observed microscopically in the interstitium of the lung and intestine 24 h after exposure to 20 Gy. Flutamide administration resulted in a similar acute inflammatory response to those noted in irradiated mice (Figure 3A &3B). More pronounced RT- induced inflammation was noted in irradiated S-mice by microscopic examination. Because TGF-β1 reportedly is an important biologic marker to predict RT-induced fibrosis [20], we further examined TGF-β1 activity by immunochemical analysis under various conditions. As shown in Figure 4, very low TGF-β1 immunoreactivities were observed in the unirradiated lung and intestinal tissues for each group. Twenty-four hours after exposure to 20 Gy, a pronounced increase in TGF-β1 immunoreactivity was observed in these tissues. A combination of irradiation with androgen deprivation by castration or Lupron Depot resulted in an increase in TGF-β1 immunoreactivity compared to radiation treatment alone, demonstrated by immunochemical staining and Western blotting (Figure 4B &4C). On the other hand, flutamide administration didn't result in the induction of increased RT-induced inflammation.

### Androgen deprivation by castration augmentation radiation-induced NF-κB activation in the lung and intestine

**Figure 5:**
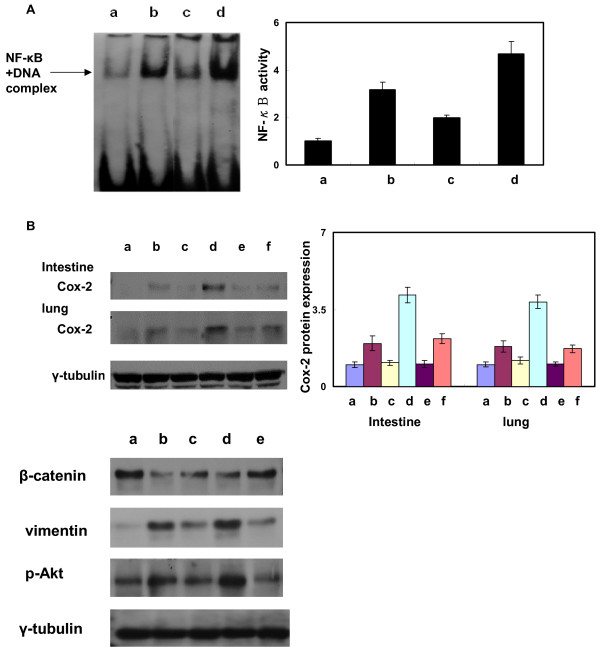
**Activation of NF-κB in murine lung and intestinal tissues**. (A) The activation of NF-κB was detected by EMSA in intestinal tissues. Representative figures are shown for (a) unirradiated control mice, (b) irradiated mice 24 h after exposure to 20 Gy, (c) flutamide-treated mice 24 h after exposure to 20 Gy and (d) castrated mice 24 h after exposure to 20 Gy. These findings demonstrate that there was significant attenuation of RT-induced the binding activity of nuclear NF-κB in castrated mice, whereas androgen deprivation by flutamide did not induce NF-κB activation. (Y axis represents the relative level which is normalized by the level of NF-κB activity in control condition). (B) Expression of COX-2 protein in irradiated murine intestinal tissue with or without androgen deprivation including castration and flutamide administration. (a, control; b, irradiation; c, castration alone; d, castration plus irradiation; e, flutamide administration; f, irradiation plus flutamide administration). Triplicate experiments were performed for the analysis. (Y axis represents the relative protein level which is normalized by the protein level of COX-2 in control condition). (C) Expression of β-catenin, vimentin and p-Akt in the intestinal tissues after various treatments (a, control; b, treated with TGF-β1; c, treated with TGF-β1+ wortmannin; d, irradiated tissue 24 h after 20 Gy; e, after pretreatment with wortmannin for 12 h followed by 20 Gy irradiation). Triplicate experiments were performed for the analysis.

NF-κB plays an important role in RT-induced inflammation [21]. To clarify whether the mechanisms responsible to the augmentation of RT-induced inflammation by castration, but not by administration of flutamide is related to the activation of NF-κB, EMSA was performed on tissues following various treatments. As shown in Figure 5A, exposure to 20 Gy promoted NF-κB binding in the lung and intestine, compared to that observed in unirradiated murine tissues. Flutamide administration appeared the tendency to attenuate the binding activity of NF-κB. However, exposure to 20 Gy in castrated mice significantly promoted NF-κB binding. We further examined the expression of COX-2 (Figure 5B), which is one of the main down-stream genes of NF-κB to induce inflammatory response.

We hypothesized that the EMT induced by TGF-β1 might contribute to the RT-induced fibrosis. To test this hypothesis, we used western blot analysis to compare p-Akt and β-catenin levels in irradiated mrine intestine with different treatments. Increased p-Akt and decreased β-catenin levels were observed after stimulation by TGF-β1 or irradiation compared to controls. Moreover, inhibition of Phosphoinositide-3 kinase by wortmannin was associated with attenuation the increased p-Akt and vimentin and decreased β-catenin stimulated by irradiation and TGF-β1 (Figure 5C).

## Discussion

Radiation pneumonitis and radiation enteritis are two of the most significant complications associated with chest and abdomen-pelvic irradiation, respectively. RT-induced production of pro-inflammatory cytokines including IL-1β, TNF-α, TGF-β1 and IL-6 have been shown to contribute significantly to the complications associated with radiotherapy [4,20,22,23]. Early overproduction of both pro-inflammatory IL-1 and TNF-α and pro-fibrogenic TGF-β1 during radiotherapy in animal studies suggests a role in the development of acute and late radiation toxicities [3]. In humans, some clinical series have shown changes in the plasma concentrations of TGF-β1 and IL-6 during radiotherapy suggesting that these variations could identify patients at risk of radiation pneumonitis [24-26]. Such data indicate that the RT-induced response *in vivo *is associated with increased expression and activity of inflammatory cytokines.

In the present study, we demonstrated that the RT-induced inflammatory response involved increased expression of the pro-inflammatory cytokines IL-1α/β, TNF-α, TGF-β1 and IL-6 and increased MPO activity, consistent with previous studies [22,23,27]. We found that androgen deprivation by castration significantly augmented the RT-induced inflammatory response in the lung and intestine, but not in the liver. The discrepancy between the modulating effects on the lung, intestine and liver might be due to organ specificity.

TGF-β1 is autoinductive and chemotactic to monocytes and macrophages and may lead to increased growth factor expression at the site of injury [28,29]. In addition, TGF-β1 is a potent chemoattractant for fibroblasts and triggers the expression of extracellular matrix components in fibrosis. Rube et al. [29] have suggested that the localization of TGF-β1 indicates areas with inflammatory cell infiltrates that are involved in the pathogenesis of RT-induced fibrosis. In the present study, we show that androgen deprivation by castration (surgical and chemical with Lupron Depot) increased TGF-β1 immunoreactivity. To examine further whether the pro-inflammatory effect of castration was related to androgen deprivation by androgen receptor blockade, we examined inflammation and TGF-β1 immunoreactivity in irradiated mice with or without flutamide administration. However, androgen deprivation by flutamide, a blocker of androgen receptor, did not augment RT-induced inflammation. Therefore, we propose there should be other mechanisms are responsible for the augmented pro-inflammatory effects in castrated mice, rather than flutamide treatment.

NF-κB is activated by many different stimuli such as radiation and oxidative stress, which induce the phosphorylation of IκB [21]. NF-κB activation is widely recognized as a key regulator of immune and inflammatory responses. Several studies have shown that NF-κB is a key transcription factor in the activation of genes encoding inflammatory cytokines including IL-1β, TNF-α, TGF-β1, IL-6 and IL-8 and induces their expression [30,31]. Thus, NF-κB is thought to have a pivotal role in the induction of cytokine expression in the inflammatory response after irradiation. We found that DNA binding activity of NF-κB after irradiation was more augmented by castration than flutamide administration. Similarly, Shimizu et al. [32] reported that the attenuation of pro-inflammatory cytokines production by flutamide is associated with inhibiting NF-κB- DNA complex. Furthermore, several studies [33,34] have reported that COX-2 is the important gene regulated by NF-κB activation and mediating the subsequent inflammation. According to Cai's report [35], combined androgen blockade induced the increased COX-2. In the present study, the increased NF-κB binding in castrated mice was associated with the more induction of COX-2 expression, in contrast to flutamide treatment. Although the mechanism responsible for this response still requires further elucidation, the increased NF-κB binding may at least partially explain why androgen deprivation by castration modulated RT-induced inflammation.

Radiation-induced fibrosis is an untoward effect of high dose therapeutic and inadvertent exposure to ionizing radiation. TGF-β is the master switch cytokine, which once activated after radiation promotes a train of cellular events that result in radiation-induced fibrosis [20,36]. Moreover, TGF-β1 was reported to play an important role in the induction of epithelial-mesenchymal transition (EMT) [24-28]. Several studies have reported that TGF-β1 induces EMT via the PI3K/Akt signaling pathways [37,38], which are associated with loss of β-catenin, a hallmark of EMT. In this study, decreased β-catenin with concurrent increases in vimentin and p-Akt were observed in murine intestine with TGF-β1 treatment, similar to that induced by irradiation. Moreover, pretreatment with wortmannin, which is a general inhibitor of PI3K family proteins, led to attenuate the increased p-Akt and vimentin and decreased β-catenin stimulated by irradiation and TGF-β1. Based on these findings, we propose that induction of EMT by TGF-β1 via the PI3K/Akt signaling pathway may contribute at least partially to RT-induced fibrosis.

## Conclusion

In summary, we show that androgen deprivation by castration, rather than flutamide administration, augmented the RT-induced inflammatory response. In contrast to flutamide, the increased NF-κB activity and subsequent elevated COX-2 by castration might be the underlying mechanism responsible to the increase in RT-induced inflammatory response. Our data also indicate that RT-induced fibrosis is related to TGF-β1-induced EMT and is probably mediated via the PI3K/Akt signaling pathway. These results suggest that sex differences play an important role in the inflammatory response. When androgen deprivation is concurrently used with irradiation treatment, the modulating effects on the RT-induced inflammation and fibrosis should take into consideration for complications associated radiotherapy. In future, we will further investigate the modulating effect of androgen on the RT- induced chronic toxicity with longer follow- up.

## Competing interests

There is no conflict of interest that could be perceived as prejudicing the impartiality of the research reported

## Authors' contributions

CTW performed the study and drafted the manuscript. WCC conceived part of the study and performed the statistical analysis. PYL helped in histology and IHC staining. CTY conceived of the study and participated in its design and coordination. MFC conceived of the study, participated in its design and coordination and assisted in editing of manuscript. All authors read and approved the final manuscript.

## Pre-publication history

The pre-publication history for this paper can be accessed here:

http://www.biomedcentral.com/1471-2407/9/92/prepub
